# Timosaponin AIII mitigates zearalenone-induced kidney injury in weaned piglets in association with TLR/MAPK signaling suppression

**DOI:** 10.3389/fvets.2026.1941423

**Published:** 2026-09-30

**Authors:** Zengxin Lv, Yijie Qin, Yingxuan Duan, Xin Liu, Yongpeng Guo, Zhixiang Wang, Wei Zhang

**Affiliations:** College of Animal Science and Technology, Henan Agricultural University, Zhengzhou, China

**Keywords:** inflammatory-apoptosis, kidney injury, piglets, Timosaponin AIII, TLR/MAPK pathway, zearalenone

## Abstract

**Introduction:**

Zearalenone (ZEN) is a common Fusarium mycotoxin that frequently contaminates feed and can induce kidney dysfunction in pigs. Timosaponin AIII (TSA), a steroidal saponin isolated from Anemarrhena asphodeloides Bunge, possesses anti-inflammatory and antioxidant properties. This study evaluated whether TSA supplementation alleviates ZEN-induced kidney injury in weaned piglets and examined its association with TLR4/MAPK signaling.

**Methods:**

Eighteen weaned female piglets were randomly assigned to three groups (*n* = 6 per group): Control (basal diet), ZEN (basal diet supplemented with 2 mg/kg ZEN), and ZEN + TSA (basal diet supplemented with 2 mg/kg ZEN and 20 mg/kg TSA). After 28 days, growth performance and kidney index were recorded. Serum CRE, BUN, and UA concentrations were measured. Kidney histopathology was assessed by H&E staining, and apoptosis was evaluated by TUNEL staining. Renal cytokines (IL-1β, IL-6, IL-10, and TNF-α) were quantified by ELISA. Expression of TLR/MAPK signaling-related genes and proteins was analyzed by qPCR and Western blot. Western blot densitometry used three biological replicates per group (*n* = 3), whereas ELISA, qPCR, serum biochemistry, and TUNEL quantification used all six animals per group (*n* = 6).

**Results:**

ZEN exposure significantly reduced final body weight, ADFI, ADG, and kidney index, and increased F/G, serum CRE and BUN concentrations, and renal histopathological lesions (*P* < 0.05). ZEN also elevated renal IL-1β, IL-6, TNF-α, and IL-10 levels and increased TUNEL-positive apoptotic cells. TSA co-administration significantly ameliorated ZEN-induced growth impairment, kidney dysfunction, histopathological damage, cytokine elevation, and apoptosis (*P* < 0.05). At the molecular level, ZEN upregulated TLR4, NF-κB, IL-1β, and JNK expression, while TSA co-administration was associated with reduced IL-1β, JNK, and p-JNK/total JNK ratio. However, the difference in TLR4 protein abundance between the ZEN and ZEN + TSA groups was not statistically significant, and MAP3K7 remained elevated while IL-1R1 was not restored, indicating selective modulation of downstream MAPK effectors rather than uniform inhibition of the entire pathway.

**Discussion:**

TSA supplementation is associated with the alleviation of ZEN-induced kidney injury in weaned piglets, concomitant with reduced inflammatory and apoptotic responses and selective modulation of TLR/MAPK downstream signaling, particularly JNK. However, due to the absence of a TSA-only control group, the lack of analytical verification of dietary ZEN and TSA concentrations, and the absence of direct pathway inhibitor, knockdown, or rescue experiments, these findings are correlative and cannot establish causality. Further studies incorporating TSA-only groups, verified feed concentrations, and pathway-specific interventions are needed to confirm the protective mechanism and safety of TSA.

## 1 Introduction

Zearalenone (ZEN) is a Fusarium-derived mycotoxin commonly detected in feed ingredients, and exposure to ZEN has been associated with multiple toxic effects that impair animal health and productivity (1). In addition to its parent form, ZEN frequently co-occurs with its derivatives, including α/β-zearalenol and α/β-zearalanol, as well as other trichothecenes, such as deoxynivalenol, thereby increasing the toxicological burden on livestock (2). Chronic dietary exposure to ZEN is well documented to induce reproductive dysfunction; it also compromises growth performance and feed efficiency in weaned piglets (3). Beyond its classical estrogenic effects, ZEN poses multisystemic risks, including hepatotoxicity, immunotoxicity, and, notably, nephrotoxicity (4–6). As a primary filtration and excretory organ with high blood perfusion, the kidney is highly susceptible to the accumulation of exogenous xenobiotics (7). Such accumulation typically triggers inflammatory responses and programmed cell death, ultimately resulting in structural and functional kidney impairment (8, 9). Despite these findings, the precise molecular mechanisms underlying ZEN-induced nephrotoxicity remain incompletely defined.

Inflammatory infiltration and apoptosis are pivotal pathological hallmarks of ZEN-mediated tissue damage (10). Activation of Toll-like receptor (TLR) signalin is closely associated with the initiation of innate immune and inflammatory responses (11). Upon activation, MyD88-dependent NF-κB signaling promotes the expression of TNF-α and IL-1β (12, 13). Current evidence indicates that ZEN aberrantly engages the TLR signaling axis, thereby intensifying kidney inflammation (14, 15). Furthermore, activation of the IL-1/IL-1R1 signaling axis is closely associated with innate immune dysregulation and the progression of inflammation-related cell death (16). Therefore, we further examined the role of MAPK signalin, which serves as a convergent downstream node for inflammatory and apoptotic signaling cascades, especially the JNK-mediated apoptotic branch, to better delineate the protective mechanism of TSA. The MAPK cascade examined here includes MAP3K7, JNK, and related molecules (17) and is linked to apoptosis and DNA damage responses (18). Crosstalk between TLR and the MAPK signaling pathway (19) may further amplify inflammatory responses and promote apoptosis through downstream kinases such as JNK and related transcriptional regulators (20). However, the involvement of the TLR/MAPK axis in ZEN-induced apoptosis in porcine kidney remains poorly characterized.

Traditional Chinese medicine-derived compounds have shown diverse biological activities, particularly regulating of inflammation, oxidative stress, and apoptosis (21). Anemarrhena asphodeloides Bunge is rich in steroidal saponins, among which Timosaponin AIII (TSA) is a major active component (22). TSA is a natural compound with multiple bioactivities, including anti-inflammatory, anticancer, and immunomodulatory effects, and it may modulate apoptosis through the MAPK pathway (23). It attenuates inflammatory responses and apoptosis by regulating TLR-related inflammatory signaling pathways (24). Timosaponin BII exerts protective effects against intestinal injury and inflammation by inhibiting NLRP3 inflammasome activation in colonic tissues and macrophages (25). The cardioprotective effects of Timosaponin BII have been linked to activation of the PI3K/Akt pathway and attenuation of endoplasmic reticulum stress induced apoptotic responses (26). Despite these observations, it remains unclear whether TSA mitigates ZEN-induced nephrotoxicity through suppression of TLR/MAPK signaling.

Accordingly, this study investigated the potential protective effects of TSA in weaned piglets exposed to ZEN. By assessing growth performance, kidney histopathology, and functional indices, we aimed to elucidate the underlying mechanisms, with a focus on the TLR/MAPK signaling axis and associated apoptotic events. These findings may help clarify the mechanisms involved in mitigating mycotoxicosis and support TSA as a targeted nutritional strategy for enhancing porcine health and productivity.

## 2 Materials and methods

All animal experimental procedures were reviewed and approved by the Animal Ethics Committee of Henan Agricultural University (Approval No. HNND2024031234). The procedures were conducted in accordance with the Guide for the Care and Use of Laboratory Animals. All weaned piglets were managed according to standardized animal welfare and ethical guidelines to minimize pain and distress.

### 2.1 Reagents

ZEN (≥98% purity) was purchased from Shanghai Yuanye Biotechnology Co., Ltd. (Shanghai, China), and TSA was obtained from ChemFaces (Wuhan, China).

### 2.2 Animal management and experimental design

A total of 18 weaned female piglets (Duroc × Landrace × Yorkshire) were obtained from Henan Anfei Animal Husbandry Technology Co., Ltd. (Henan, China). The company, as the owner of the experimental animals, provided written approval for their use in the present study prior to the start of the experiment. The piglets were randomly assigned to three experimental groups (*n* = 6 per group): Control (basal diet), ZEN (basal diet supplemented with 2 mg/kg ZEN), and ZEN + TSA (basal diet supplemented with 2 mg/kg ZEN and 20 mg/kg TSA). TSA powder was thoroughly mixed with the basal diet before feeding. For ZEN administration, ZEN was dissolved in anhydrous ethanol at a 1:1 (w/v) ratio 1 day before feeding, and the solution was evenly sprayed onto a portion of the diet with a spray bottle. Confirm that residual ethanol was not present in the final feed. After complete ethanol evaporation, the contaminated feed was thoroughly blended with the basal diet to ensure uniform ZEN distribution. The control group and the TSA + ZEN group received identical ethanol treatment. All piglets were housed individually under identical environmental conditions, with controlled room temperature and continuous ventilation for 24 h per day. On day 29 of the experiment, growth performance parameters were recorded, and all animals were subsequently euthanized for sample collection.

### 2.3 Sample collection

After the 28-day experimental phase, the final body weight of each piglet was recorded before humane euthanasia and tissue collection. Humane euthanasia was performed via intravenous injection of an overdose of pentobarbital sodium solution (40 mg/kg body weight). All animal carcasses were finally sterilized by autoclaving for harmless disposal. Whole blood samples were collected and promptly centrifuged to separate serum for downstream biochemical analyses. Kidney tissues were carefully excised, weighed, and divided for subsequent assays. Specifically, kidney tissues were either fixed in 4% paraformaldehyde for histological analysis or snap-frozen in liquid nitrogen for molecular and biochemical analyses. Blood samples were obtained from all piglets by anterior vena cava puncture prior to euthanasia. The collected whole blood was left to clot at room temperature (25 °C) for 30 min and centrifuged at 3,500 rpm for 15 min. The serum (pale yellow supernatant) was carefully collected into sterile 1.5-mL tubes, avoiding contamination with red blood cells and hemolysis. Fresh serum was used immediately for biochemical assays, while the remaining samples were aliquoted and frozen at −80 °C for cytokine analysis ELISA. Freeze–thaw cycles were avoided. Kidney tissues were fixed in 4% paraformaldehyde for 48 h at room temperature, dehydrated in graded ethanol, and embedded in paraffin. The interval between fixation and embedding was kept within 72 h.

### 2.4 Diet composition

The basal diet was formulated to meet the Chinese national standards (GB) for weaned piglet nutrition. A comprehensive breakdown of feed ingredients and nutrient profiles is provided in Supplementary materials 1. ME was calculated based on the inclusion rate of each feed ingredient and its corresponding reference metabolizable energy value. CP was determined by the Kjeldahl method in accordance with GB/T 6432. Ca and TP were analyzed in accordance with GB/T 6436 and GB/T 6437, respectively. AP was determined using hydrochloric acid extraction and spectrophotometry in combination with GB/T 6437. The levels of the amino acids Lys, Met, and Thr were determined by HPLC in accordance with GB/T 18246. Additionally, we measured the ZEN concentrations in the diets of the three experimental groups. The zearalenone concentration in the control group diet was 79.01 ppb, in the ZEN group diet it was 2060.78 ppb, and in the ZEN + TSA group diet it was 2109.90 ppb.

### 2.5 Growth performance and kidney index

ADG, ADFI, and F/G were calculated to evaluate growth performance. During sample collection, kidneys tissues were carefully excised, and the surrounding adipose tissue was thoroughly removed before weighing. Fresh kidneys weighed with an electronic balance before downstream evaluations. The relevant calculations were performed according to the following equations:


$$\mathrm{ADG}\;(g/d)=(\begin{array}{l} \text{Final body weight}-\\ \text{Initial body weight} \end{array})/\text{Experimental duration}$$

ADFI(g/d)=Total feed intake/Experimental duration

F/G=ADFI/ADG

$$\begin{array}{l} \text{Kidney index}\;(g/\mathrm{kg})=\text{Fresh kidney weight}\;(g)/\\ \text{Live body weight}\;(\mathrm{kg}) \end{array}$$

### 2.6 Levels of inflammatory cytokines in kidney tissue

ELISA kits for IL-1β, IL-6, IL-10, and TNF-α were purchased from Shanghai Jianglai Biotechnology Co., Ltd. (Shanghai, China). Kidney tissue samples were homogenized and subsequently subjected to the bicinchoninic acid (BCA) assay to determine protein concentratio. Intrarenal levels of key pro- and anti-inflammatory cytokines (IL-1β, IL-6, TNF-α, and IL-10) were quantified using commercial ELISA kits in strict accordance with the manufacturer’s protocols.

### 2.7 Histopathological observation of kidney tissue

To ensure anatomical consistency, kidney were collected from the same region in each. The specimens were immediately preserved in 4% paraformaldehyde before routine histological processin including paraffin mbedding, microtome sectioning, and standard hematoxylin and eosin (H&E) staining for histopathological examination. The stained sections were scanned and imaged using a KF-FL-400 digital slide scanner (Jiangfeng Biotechnology, Ningbo, China). Histopathological alterations in kidney tubules and glomeruli were observed using CaseViewer software (3DHISTECH Ltd., Budapest, Hungary), and the extent of kidney injury was further evaluated based on the observed morphological changes.

### 2.8 Determination of serum kidney function parameters

Serum BUN, UA, and CRE concentrations were determined using an automatic biochemical analyzer (BS-60S, Mindray Bio-Medical Electronics Co., Ltd., Shenzhen, China).

### 2.9 Total RNA extraction and qPCR analysis

Total RNA was extracted from porcine kidney tissues using an RNA extraction kit (D9108B, TaKaRa, Japan) according to the manufacturer’s protocol (27). RNA quantity and purity were assessed from A260/280 ratios using a NanoDrop One/OneC spectrophotometer (P800, Implen, Germany). Subsequently, cDNA was synthesized from total RNA using a commercial reverse -transcription kit according to the manufacturer’s instructions. The primer sequences used in the present study are listed in Table 1 and were synthesized by Sangon Biotech (Shanghai, China). Quantitative real-time PCR (qPCR) was performed to determine gene expression levels. The 2^^−ΔΔCt^ method was used to calculate relative mRNA expression levels. β-actin was used as the internal reference gene for normalization. Reagents used for RT-qPCR analysis were supplied by Takara Biomedical Technology Co. (Beijing, China). Gene sequences obtained from NCBI served as templates for primer design, and primer synthesis was performed by Sangon Biotech (Shanghai, China) (28).

**Table 1:** Primer sequences of genes associated with inflammatory and apoptotic signaling pathways.

Gene	Sequence (5′-3′)		Product size (bp)
TLR4	F:	GGACCTGCCTGTGCTGAGTTTC	126
	R:	TGCCACAGATACCAGGAGGAAGG	
NF-κB	F:	CCGTGTCTGCTGCTGCTGATG	99
	R:	GCCCGCCAAGGAGATGTTGTC	
IL-1β	F:	GCCAACGTGCAGTCTATGGAGTG	112
	R:	GGTGGAGAGCCTTCAGCATGTG	
TNF-α	F:	GCACTGAGAGCATGATCCGAGAC	120
	R:	CGACCAGGAGGAAGGAGAAGAGG	
IKBKB	F:	ACCTTGGCCTTTGAGTGCAT	145
	R:	AGCTGGAGAACTTCACGGTG	
TIRAP	F:	TCCTCGCTCCAAAAAGCCTT	81
	R:	ACATGAGTCGTTGTGGGCTT	
IL-1R1	F:	CCGCACACAATGGGCACTATACC	146
	R:	CCATCGTCTCATTGGCTGGACTG	
MAP3K7	F:	GGAGCCTGCTTGAATCCAGT	175
	R:	AAGCTTTGGGTTGCATGCTG	
JNK	F:	TCGCTACTACAGAGCACCTGAGG	86
	R:	CTCCCATAATGCACCCCACAGAC	
JUND	F:	AGTCCAACGGGCTGGTCACC	88
	R:	TCGGCGAACTCCTGCTCCTC	
β-actin	F:	CTACCTCATGAAGATCCTGACC	90
	R:	CACAGCTTCTCTTTGATGTCAC	

### 2.10 Extraction of kidney tissue proteins and Western blot analysis

Kidney tissues were stored at −80 °C until use. Kidney tissues were homogenized in protein extraction buffer, and protein concentrations were determined using a BCA-based method. We added protease and phosphatase inhibitors to the protein lysis buffer for every sample during the extraction process. Protein samples were mixed with loading buffer, heat-denatured at 100 °C for 10 min, separated by SDS–PAGE, and subsequently transferred onto PVDF membranes. After blocking with 5% skim milk for 2 h at room temperature, the membranes were incubated with primary antibodies overnight at 4 °C and then with the corresponding secondary antibodies for 1 h at room temperature (29). For WB analysis, protease inhibitors used in this study were provided by Solepol Biotechnology Co., Ltd. (Beijing, China). The prestained protein marker (WJ103), TBS/Tween buffer (PS103S), electrophoresis buffer (PS105S), and transfer buffer (PS109S) were all purchased from Yase Biomedical Technology Co., Ltd. (Shanghai, China). The primary antibodies used for Western blot analysis are shown in Supplementary materials 1.

### 2.11 TUNEL analysis of cell apoptosis

After deparaffinization and rehydration, sections were rinsed with PBS and permeabilized for 8–10 min. A 50 μL TdT reaction mixture was applied, followed by incubation at 37 °C for 1 h in the dark. After washing, TUNEL-positive cells were identified by their green fluorescence under a fluorescence microscope. During data processing, a single-blind method was employed for the analysis of fluorescent sections. We also confirm that one section and three high-power fields (HPFs) were obtained from each animal.

### 2.12 Statistical analysis

The applicability of parametric statistical methods was verified by assessing data distribution and homogeneity of variance with Shapiro–Wilk and Levene’s tests, respectively. Statistical analyses were performed using SPSS Statistics 26.0 (IBM Corp., Armonk, NY, USA). Results are presented as the mean ± SEM, and statistical significance was set at *p* < 0.05. One-way ANOVA was used for comparisons among multiple groups, followed by Tukey’s HSD *post hoc* test. The analyzed parameters included growth performance, kidney index, serum biochemical parameters, ELISA-determined inflammatory cytokines, inflammation- and apoptosis-related proteins, and Western blot densitometric data. Graphical presentations were generated using GraphPad Prism 9.0 (GraphPad Software Inc., San Diego, CA, USA).

## 3 Results

### 3.1 Effects of ZEN and TSA on growth performance in piglets

As shown in Table 2, compared with the CON group, piglets in the ZEN-treated group exhibited significantly reduced final body weight on day 28, ADFI, and ADG (*p* < 0.05), whereas F/G was significantly increased (*p* < 0.05). In contrast, TSA supplementation markedly alleviated these adverse effects of ZEN exposure. Final body weight, ADFI, ADG, and F/G in the ZEN + TSA group were restored to levels comparable to those observed in the CON group.

**Table 2:** Effects of ZEN and TSA on growth performance in piglets.

Items	CON	ZEN	ZEN+TSA	SEM	*p*-value
Initial body weight (kg)	6.74	6.78	6.93	0.240	0.775
Final body weight (kg)	16.82^a^	13.89^b^	16.64^a^	0.473	0.005
ADFI (g/d)	557.31^a^	453.42^b^	544.13^a^	14.016	<0.001
ADG (g/d)	326.59^a^	262.84^b^	322.18^a^	8.823	<0.001
F/G	1.75^b^	1.97^a^	1.76^b^	0.043	0.045

a,b significantly different (*p* < 0.05).

### 3.2 Histopathological observation of the effects of ZEN and TSA on kidney injury in piglets

As shown in Figure 1, the CON group exhibited normal kidney architecture, characterized by intact glomerular structures and well-defined kidney tubules, without apparent lesions. In contrast, ZEN exposure induced distinct histopathological alterations, including marked tubular dilation and multifocal inflammatory infiltration within the interstitium and some glomeruli. TSA intervention substantially restored glomerular and tubular morphology, with and the kidney microarchitecture in the ZEN + TSA group appeared comparable to that of the CON group.

**Figure 1:**
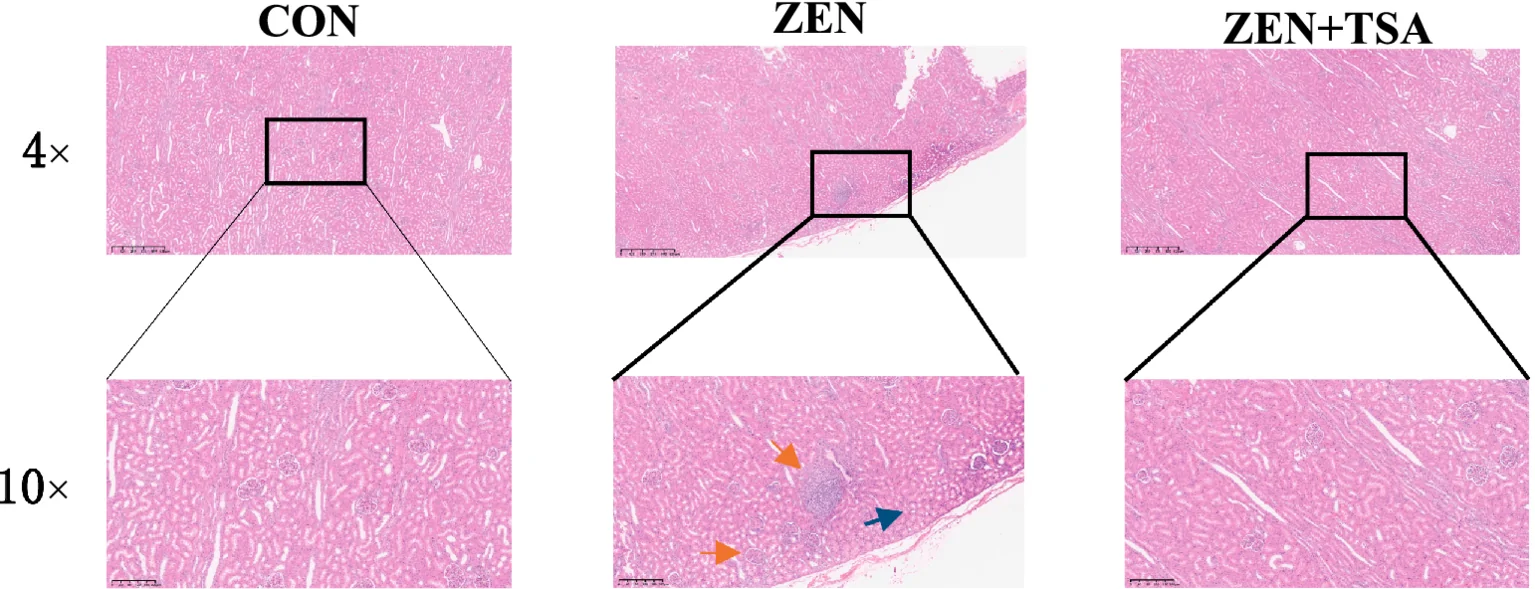
Histopathological observations of the effects of ZEN and TSA on kidney injury in piglets. Representative images are shown at 4× and 10× magnification. Blue arrows indicate abnormal dilation of kidney tubules, whereas orange arrows indicate foci of inflammatory infiltration within glomeruli. (Scale bar = 200 μm).

### 3.3 Effects of ZEN and TSA on serum kidney function parameters in piglets

As shown in Figure 2, compared with the CON group, the ZEN-treated group showed significantly elevated serum CRE and BUN concentrations (*p* < 0.05). TSA supplementation markedly reversed these changes, with serum CRE and BUN concentrations in the ZEN + TSA group returning to levels comparable to those in the CON group (*p* < 0.05). Serum UA concentrations remained comparable across all groups (*p* > 0.05).

**Figure 2:**
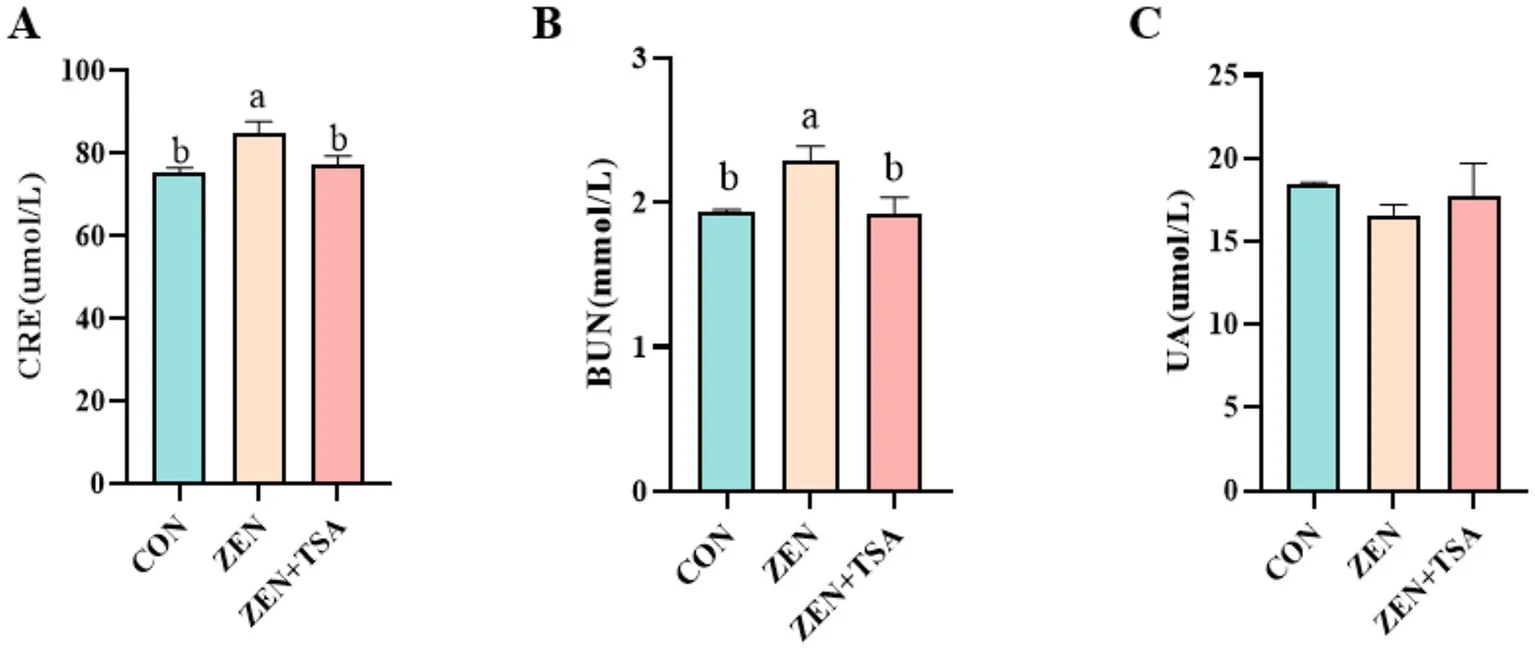
Effects of ZEN and TSA on serum kidney function parameters in piglets. **(A)** CRE; **(B)** BUN; **(C)** UA. Data are presented as the mean ± SEM (*n* = 6). Means bearing different lowercase letters are significantly different (*p* < 0.05).

### 3.4 Effects of ZEN and TSA on kidney index and kidney inflammatory cytokine levels in piglets

As shown in Figure 3A, the kidney index was significantly decreased in the ZEN-treated group compared with the CON group (*p* < 0.05). However, TSA supplementation markedly increased the kidney index in the ZEN + TSA group compared with the ZEN group (*p* < 0.05), indicating that TSA partially alleviated ZEN-induced kidney impairment. As shown in Figure 3B, ZEN exposure significantly increased the concentrations of IL-1β, IL-6, IL-10, and TNF-α in kidney tissues (*p* < 0.05). In contrast, TSA supplementation markedly reversed these alterations, and the levels of IL-1β, IL-6, IL-10, and TNF-α in the ZEN+ + TSA group were restored to levels comparable to those in the CON group (*p* < 0.05).

**Figure 3:**
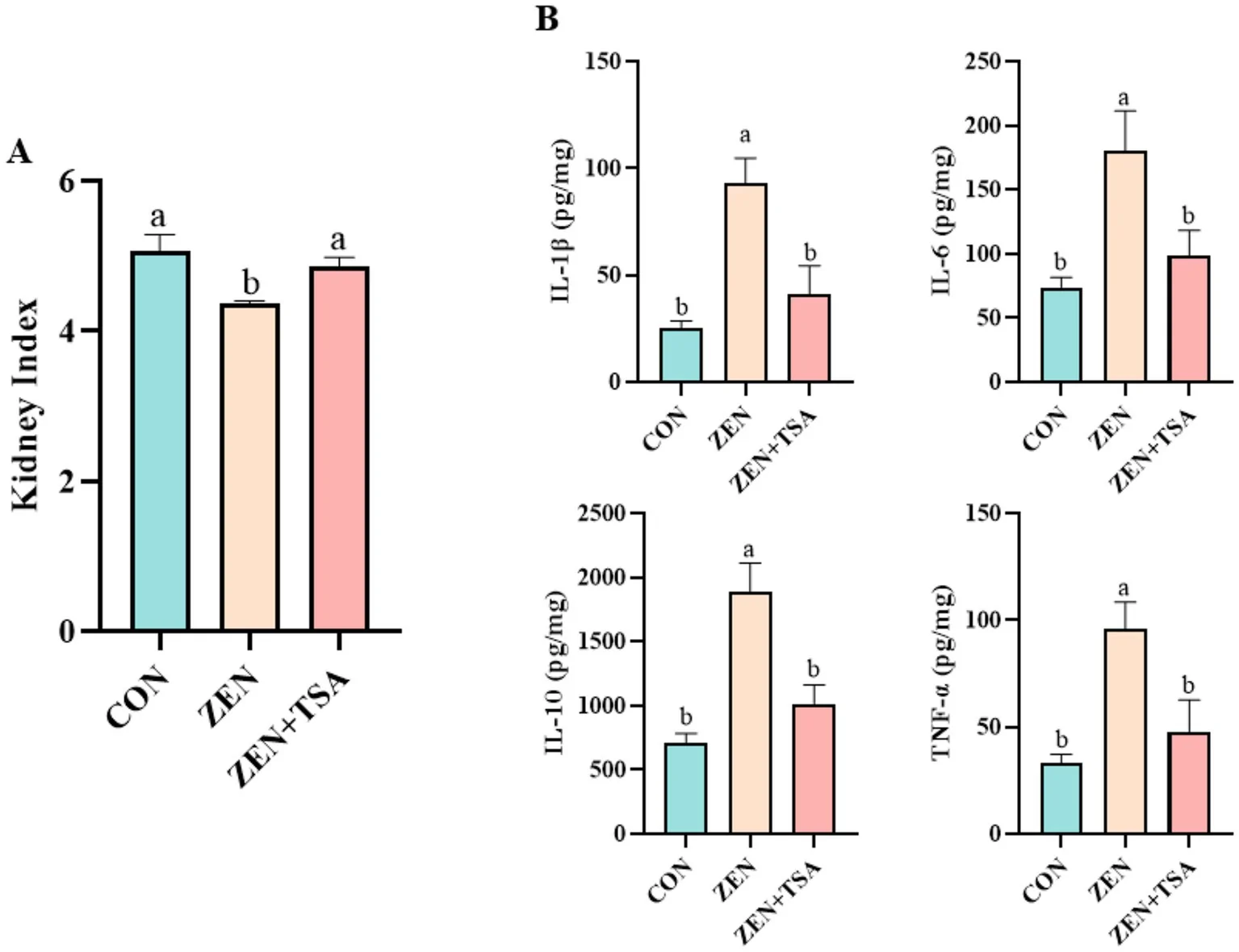
Effects of ZEN and TSA on kidney index and kidney inflammatory cytokine levels in piglets. **(A)** Kidney index (*n* = 6). **(B)** Concentrations of IL-1β, IL-6, IL-10, and TNF-α in kidney tissues measured by ELISA. Data are presented as the mean ± SEM (*n* = 6). Means bearing different lowercase letters are significantly different (*p* < 0.05).

### 3.5 Effects of ZEN and TSA on TLR signaling pathway-related gene and protein expression in kidney tissue

As shown in Figure 4A, compared with the CON group, ZEN exposure significantly upregulated the transcripts of TLR signaling–-related inflammatory genes, including TNF-α, IL-1β, NF-κB, IKBKB, TIRAP, and TLR4. (*p* < 0.05). In contrast, TSA treatment largely normalized the expression of TNF-α, IL-1β, NF-κB, IKBKB, TIRAP, and TLR4, with transcript levels comparable to those observed in the CON group (*p* < 0.05). To further validate these findings, Western blot analysis was performed. As shown in Figures 4B,C, ZEN treatment markedly enhanced NF-κB and TLR4 protein abundance (*p* < 0.05). However, TSA treatment significantly attenuated these increases, largely normalizing NF-κB and TLR4 protein abundance (*p* < 0.05). These results indicate that TSA effectively alleviated ZEN-induced kidney inflammatory responses.

**Figure 4:**
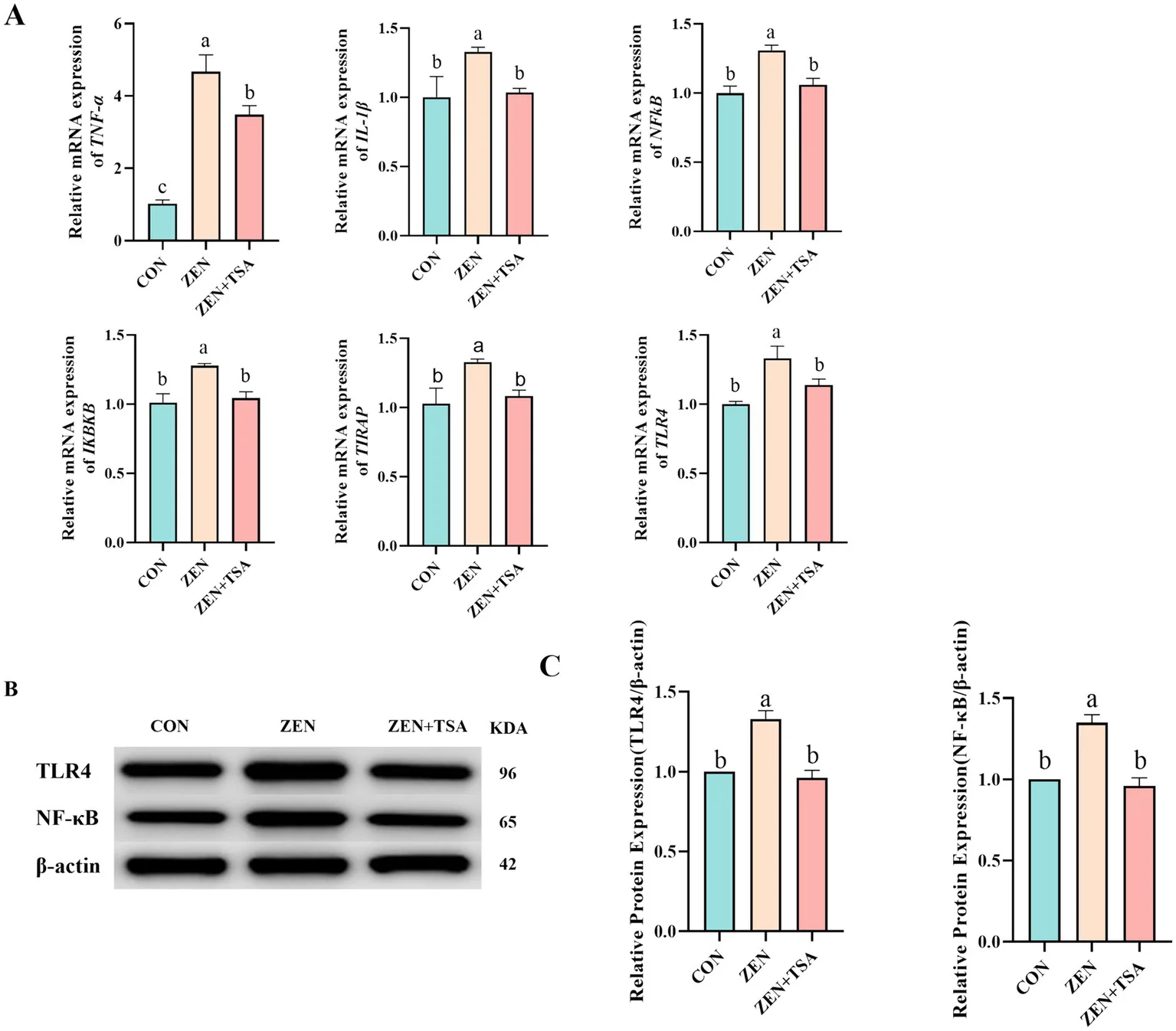
Effects of ZEN and TSA on the gene and protein expression of the kidney inflammation-related TLR signaling pathway. **(A)** Relative mRNA expression of TLR pathway–associated genes (*n* = 6). **(B)** Protein abundance of selected TLR pathway-related factors in kidney tissues following ZEN exposure and TSA treatment. Representative immunoblots from three independent experiments are shown (*n* = 3). **(C)** Densitometric quantification of the bands shown in panel B normalized to β-actin. Data are presented as the mean ± SEM. Means bearing different lowercase letters are significantly different (*p* < 0.05).

### 3.6 Effects of ZEN and TSA on kidney cell apoptosis in piglets

As shown in Figures 5A,B, compared with the CON group, the apoptotic index in kidney tissues was significantly increased in the ZEN-treated group (*p* < 0.05). However, TSA treatment significantly attenuated ZEN-induced apoptotic cell death, as shown by the marked reduction in the apoptotic index compared with the ZEN group (*p* < 0.05).

**Figure 5:**
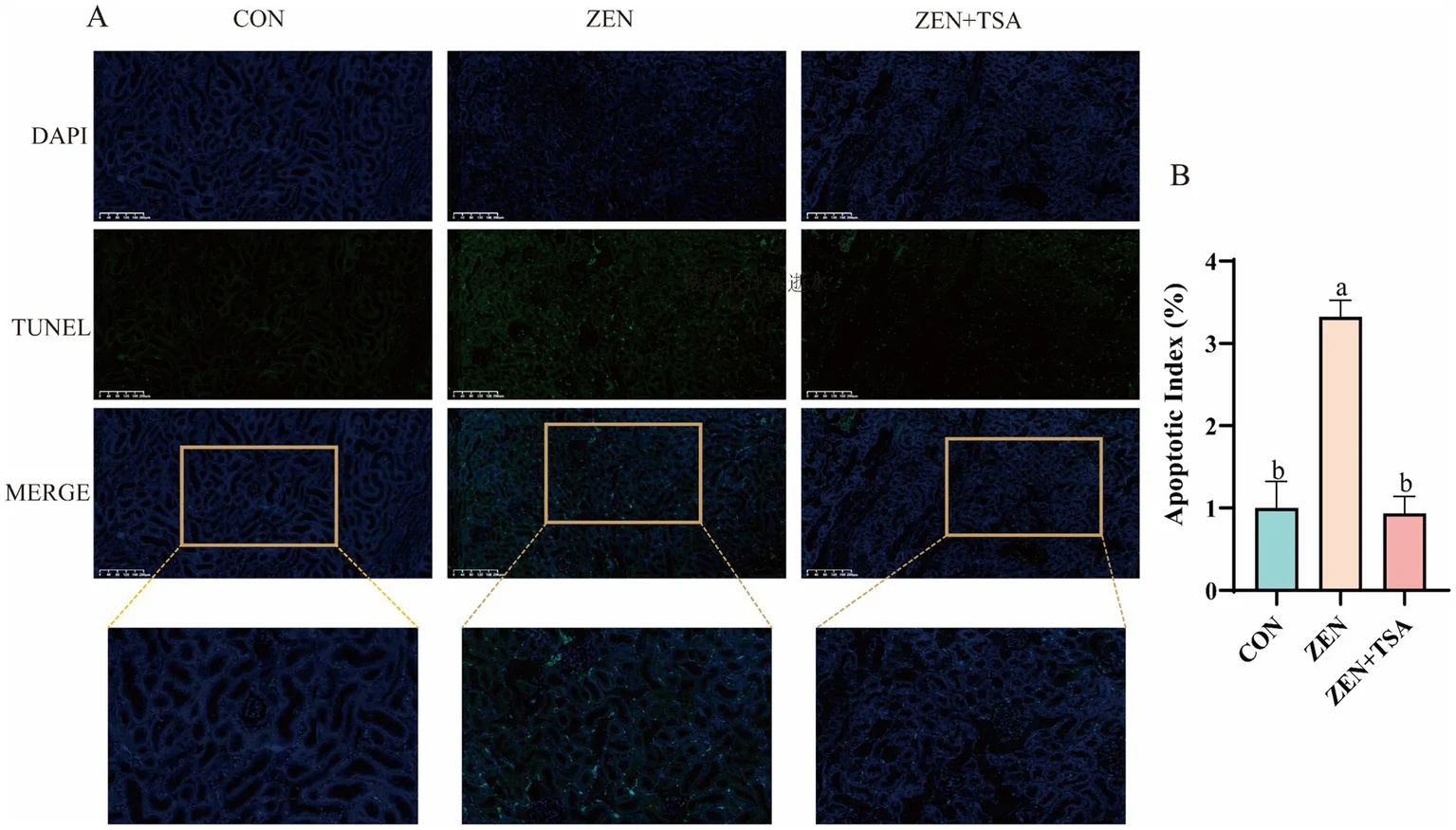
Effects of ZEN and TSA on kidney cell apoptosis in piglets. **(A)** Assessment of apoptosis in kidney tissues by TUNEL staining. **(B)** The ratio of TUNEL-positive cells to DAPI-positive nuclei in Figure A represents the apoptotic index. Data are presented as the mean ± SEM (*n* = 6). Means bearing different lowercase letters are significantly different (*p* < 0.05). (Scale bar = 200 μm).

### 3.7 Effects of ZEN and TSA on MAPK signaling pathway-related inflammatory and apoptotic gene and Protein expression in kidney tissue

As shown in Figure 6A, compared with the CON group, ZEN exposure significantly upregulated the transcripts of MAPK signaling–-related inflammatory genes, including IL-1β, MAP3K7, IL-1R1, and JNK (*p* < 0.05). In contrast, JUND expression was markedly decreased (*p* < 0.05). Compared with the ZEN group, TSA supplementation restored kidney mRNA levels of IL-1β, JNK, and JUND to levels comparable to those observed in the CON group (*p* < 0.05). Furthermore, MAP3K7 gene expression was significantly elevated after TSA treatment (*p* < 0.05), whereas IL-1R1 expression was comparable between the ZEN and ZEN + TSA groups (*p* > 0.05). To further confirm these findings, Western blot analysis was performed. As shown in Figures 6B,C, the protein abundance of IL-1β, JNK, and p-JNK was markedly increased in the ZEN group compared with the CON group (*p* < 0.05). However, TSA treatment significantly attenuated these increases, largely normalizing IL-1β, JNK, and p-JNK protein abundance (*p* < 0.05). No significant effect on the p-JNK/JNK ratio was observed. These findings indicate that TSA effectively attenuates ZEN-induced inflammation-associated apoptosis in the kidney.

**Figure 6:**
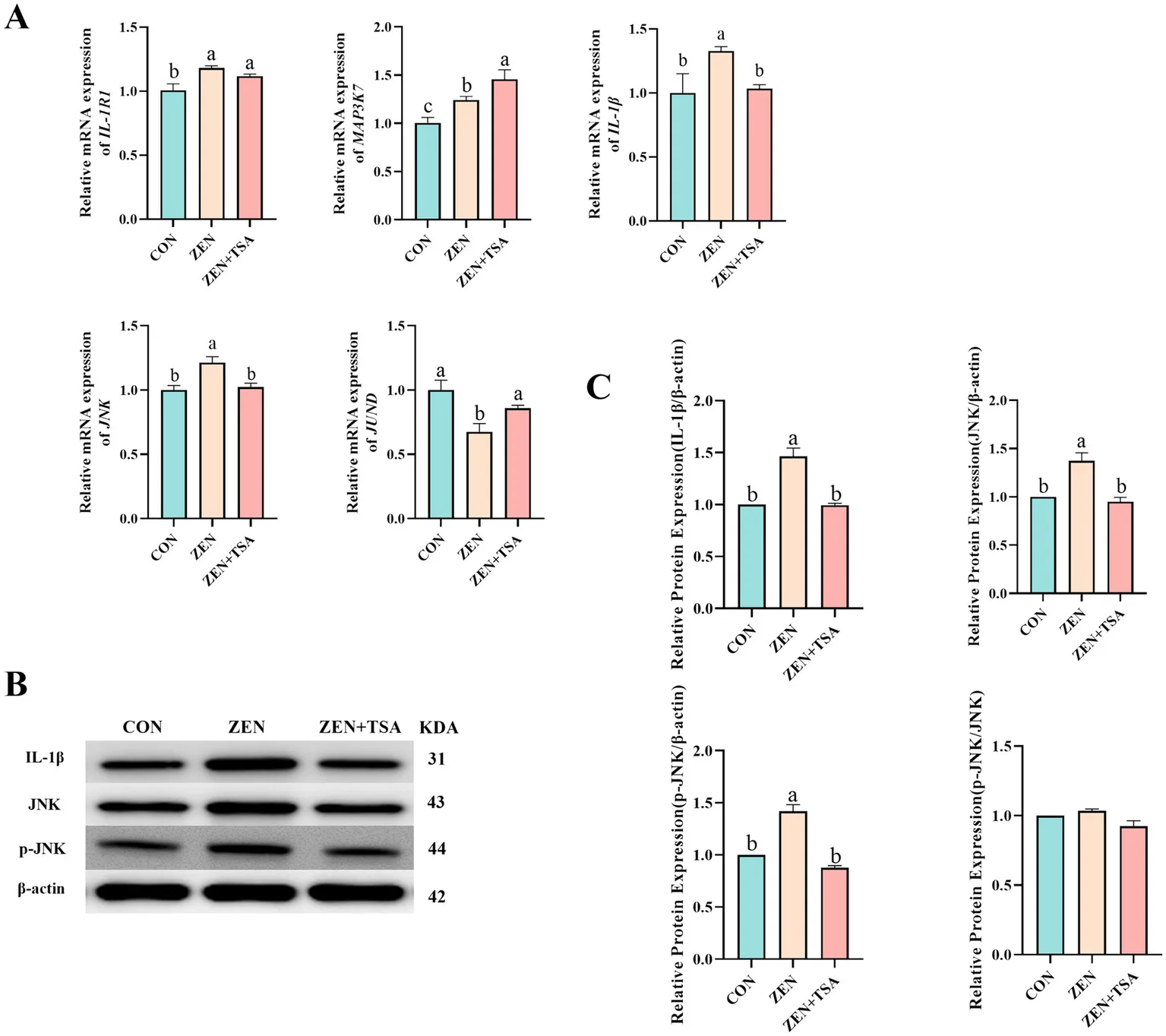
Effects of ZEN and TSA on the gene and protein expression of the kidney inflammation-associated apoptosis-related MAPK signaling pathway. **(A)** Relative mRNA expression of MAPK pathway–associated genes (*n* = 6). **(B)** Protein abundance of selected MAPK pathway-related factors in kidney tissues following ZEN exposure and TSA treatment. Representative immunoblots from three independent experiments are shown (*n* = 3). **(C)** Densitometric quantification of the bands shown in panel B was normalized to β-actin. Data are presented as the mean ± SEM. Means bearing different lowercase letters are significantly different (*p* < 0.05).

## 4 Discussion

ZEN is a white, crystalline mycotoxin distributed across many regions, including Europe, the Americas, and Asia. It is frequently found in cereal crops such as corn and wheat and in animal feed. After ingestion, ZEN has been reported to exert multiple toxic effects, including reproductive, toxicity, hepatotoxicity, gastrointestinal toxicity, and immunotoxicity, posing serious risks to animal production and human health (30). As a major excretory organ, the kidney plays a critical role in blood filtration and substance reabsorption, which predisposes it to xenobiotic accumulation and subsequent kidney injury (6). Accumulating evidence indicates that sensitivity to ZEN varies among animal species, with pigs being particularly susceptible to its toxic effects (31). The traditional Chinese medicinal plant Anemarrhena asphodeloides Bunge contains numerous secondary metabolites, including steroidal saponins, flavonoids, volatile oils, and alkaloids, which collectively exert anti-inflammatory, antidiabetic, and antitumor effects (32). Previous studies have demonstrated that TSA attenuates UVB-induced inflammatory responses and DNA damage by targeting the NF-κB/MAPK signaling pathway, further supporting its potential role in alleviating inflammation-related tissue injury (33). In the present study, ZEN exposure significantly reduced the kidney index and impaired growth performance in weaned piglets. These alterations were markedly alleviated by TSA supplementation, with the kidney index and growth -performance parameters restored to near-normal levels. Previous studies have reported that ZEN-induced reductions in the kidney index may lead to glomerular atrophy and subsequent kidney injury (34). Licorice extracts rich in saponins have also been shown to significantly alleviate ereductions in growth performance induced by exposure to ZEN and DON in broiler chickens (35), finding consistent with the current study. The marked improvement observed after TSA treatment suggests that TSA has the potential to alleviate ZEN-induced adverse effects on growth performance and kidney injury in female piglets through the TLR/MAPK signaling pathway.

Histopathological examination of H&E-stained kidney sections further demonstrated that ZEN exposure induced marked kidney lesions, including abnormal dilation of kidney tubules, intraglomerular inflammatory cell infiltration, and focal inflammatory cell infiltration. These pathological changes are consistent with those reported previously (36, 37). Importantly, No pathological changes were detected in the kidney tissue following TSA administration, particularly tubular dilation, and largely preserved the normal morphology of glomeruli and kidney tubules in piglets. These findings indicate that TSA effectively alleviated ZEN-induced kidney pathological kidney injury. Qu et al. (38) quantified serum IL-1β, IL-6, and TNF-α by ELISA and demonstrated that six ginsenosides significantly attenuated LPS-induced elevations in pro-inflammatory cytokines, thereby alleviating colitis in mice. In the present study, ELISA revealed that TSA markedly attenuated the ZEN-induced dysregulation of both pro-inflammatory (IL-1β, IL-6, and TNF-α) and anti-inflammatory (IL-10) cytokine levels in the kidney. The elevation of IL-10 might represent a compensatory anti-inflammatory mechanism. Taken together, these results demonstrate that TSA provides protection against renal inflammation triggered by ZEN.

CRE, UA, and BUN are widely recognized as important biochemical indicators of kidney function. These parameters reflect kidney filtration and reabsorption capacity, protein catabolism, nitrogenous waste excretion, and the extent of tissue injury; therefore, they are commonly used to assess the severity of kidney damage (39). Taraxasterol, a pentacyclic triterpenoid compound, has been reported to significantly attenuate the elevation of kidney injury-related parameters induced by ZEN exposure (40). In the present study, ZEN exposure significantly elevated serum CRE and BUN levels in weaned piglets, whereas these alterations were markedly reversed by TSA supplementation. The elevated CRE and BUN levels observed in the ZEN-treated group may reflect increased nitrogenous waste accumulation associated with protein catabolism and impaired glomerular filtration and kidney reabsorption capacity (41). Such kidney dysfunction may ultimately contribute to the accumulation of metabolic waste products in the bloodstream, thereby aggravating kidney injury. Consistent with previous reports, these findings indicate that ZEN exposure impairs kidney function (42). Notably, TSA treatment effectively attenuated the abnormal elevation of serum CRE and BUN induced by ZEN exposure, indicating that TSA exerts a protective effect on kidney function. The protective effects of TSA may be associated with its anti-inflammatory and cytoprotective activities, which help preserve glomerular integrity and maintain normal kidney metabolic activity under toxic stress. This interpretation is consistent with the histopathological evidence and previous findings (43). Overall, these findings demonstrate that TSA protects against ZEN-induced nephrotoxicity in weaned piglets.

The TLR signaling pathway is a key regulatory pathway in innate immune and inflammatory responses and plays a central role in the initiation and amplification of inflammation. After activation, TLRs recruit downstream adaptor proteins such as MyD88 and TIRAP, which subsequently activate NF-κB-related signaling cascades and promote the transcription and release of pro-inflammatory cytokines, including TNF-α, IL-1β, and IL-6 (44, 45). In the present study, ZEN exposure markedly activated the TLR signaling pathway, as evidenced by the significant upregulation of pathway-related mRNA and protein expression levels in kidney tissues. Notably, IL-1β expression at both the transcriptional and protein levels was significantly elevated after ZEN exposure, which may further contribute to activation of the downstream MAPK signaling pathway examined in this study. After TSA supplementation, these parameters were significantly restored toward normal levels. Resveratrol has been reported to mitigate ZEN-induced upregulation of TNF-α, IL-6, and IL-1β by regulating the TLR pathway and inhibiting NF-κB p65 nuclear translocation (46). Rutin has also been shown to mitigate ZEN-induced hepatic inflammation and injury by inhibiting IL-1β production through the TLR4/NF-κB/MyD88 signaling pathway (47). Consistent with our results, these findings suggest that ZEN-induced kidney inflammation is closely linked to TLR signaling activation, whereas TSA, a plant-derived compound, may attenuate inflammation by inhibiting this pathway.

The MAPK signaling pathway plays a pivotal role in regulating gene expression, cell cycle progression, and apoptotic processes (48). The MAPK related components examined in this study included MAP3K7/TAK1, JNK, and JUND. In immunology and inflammation studies, MAP3K7/TAK1 is generally regarded as a central downstream signaling hub for receptors such as TLRs, IL-1R, and TNF-R, mediating activation of the IKK complex and subsequent MAPK cascades. Among these signaling components, JNK and JUND are closely associated with inflammatory regulation (49). JNK activation can modulate AP-1 transcriptional activity, including JUND-related transcriptional regulation, thereby influencing genes associated with cell proliferation, differentiation, apoptosis, and inflammatory responses. In this study, ZEN exposure activated the TLR signaling pathway, leading to inflammation. Consistent with excessive IL-1β production, marked alterations in downstream MAPK pathway factors, including IL-1R1, MAP3K7, JNK, and JUND, were observed at both the mRNA level and, for selected proteins, at the protein level. These results suggest that inflammatory signaling activates downstream MAPK effectors, leading to apoptotic responses and subsequent renal injury. TSA supplementation effectively reversed most abnormal changes induced by ZEN exposure. The expression levels of IL-1β, JNK, and JUND were restored toward normal levels after TSA treatment, TSA significantly attenuated MAPK pathway signal transduction. Interestingly, the sustained elevation of MAP3K7 expression after TSA treatment may suggest that TSA participates in adaptive cellular defense responses against ZEN-induced inflammation-associated apoptosis (50). But the sustained or elevated expression of MAP3K7 in the TSA-treated group, despite reduced JNK phosphorylation, may represent an adaptive cellular feedback mechanism against ZEN-induced injury, rather than a direct pharmacological action of TSA on this upstream kinase. Furthermore, Western blot analysis of the phosphorylated form of JNK (p-JNK), a key downstream protein, confirmed that ZEN induces kidney tissue injury by activating the MAPK signaling pathway. Notably, TSA treatment significantly reduced p-JNK expression, suggesting that TSA alleviates kidney injury by suppressing excessive activation of the MAPK pathway. For example, chrysosplenol D has been reported to suppress inflammation-associated apoptosis by regulating the TLR4-MAPK/NF-κB signaling pathway (51). However, we found that the p-JNK/JNK ratio did not show significant differences among the three groups. This observation may be explained by the simultaneous increases and decreases in both p-JNK and JNK levels. Similarly, TSA significantly attenuated activation of ZEN-induced inflammatory and apoptotic signaling pathways. Moreover, TSA treatment markedly attenuated ZEN-induced apoptosis in kidney tissues, as indicated by TUNEL staining, further confirming its anti-apoptotic effects. The cumulative evidence indicates that ZEN induces kidney inflammation-associated apoptosis through activation of the TLR/MAPK signaling pathway. In contrast, TSA supplementation is associated with the alleviation of kidney injury, which correlates with reduced inflammatory and apoptotic responses and suppression of excessive TLR/MAPK signaling activation. These results further highlight the potential of TSA as a natural dietary intervention for preventing mycotoxin-induced kidney damage.

Nevertheless, the present study has several limitations. Our analysis focused solely on the TLR/MAPK pathway, without accounting for potential crosstalk from other relevant signaling cascades. Moreover, we did not use pharmacological inhibitors, genetic knockdown, or rescue approaches to directly establish the involvement of the TLR/MAPK axis in the kidney. Accordingly, future *in vivo* loss-of-function studies are necessary to conclusively confirm this causal relationship.

## 5 Conclusion

In conclusion, the present study demonstrated that TSA supplementation is significantly associated with the alleviation of ZEN-induced kidney injury in weaned female piglets, characterized by reduced inflammatory cytokine production and inflammation-associated apoptosis, alongside suppression of excessive TLR/MAPK signaling activation. Nevertheless, due to the lack of direct pathway interference experiments, these conclusions are correlative; causal inferences regarding the ‘protective mechanism’ require further experimental validation. TSA also improved kidney health by alleviating serum biochemical abnormalities and decreasing inflammatory responses in kidney tissues. These findings highlight TSA as a promising dietary strategy for preventing ZEN-induced nephrotoxicity in pigs.

## Data Availability

The original contributions presented in the study are included in the article/Supplementary material, further inquiries can be directed to the corresponding author/s.
