# Supplementary material for: Timosaponin AIII mitigates zearalenone-induced kidney injury in weaned piglets in association with TLR/MAPK signaling suppression
Source: Front Vet Sci. 2026 Sep 30;13:1941423. doi: 10.3389/fvets.2026.1941423 (PMC13663026; doi:10.3389/fvets.2026.1941423)
Supplement: Supplementary file 1 [file Supplementary_file_1.docx]

| Ingredient composition and calculated nutrient levels of the basal diet (%) | | | |
| --- | --- | --- | --- |
| **Ingredient (%)** | **Basal diet** | **Nutrients** | **Basal diet** |
| Corn | 42.05 | Metabolizable energy ^1^(MJ/kg) | 14.68 |
| Wheat | 20.00 | CP^2^(%) | 18.59 |
| Corn germ meal | 3.00 | Ca^2^(%) | 0.70 |
| Soybean meal (46% crude protein) | 4.30 | TP^2^(%) | 0.61 |
| Soy protein concentrate | 7.08 | AP^2^(%) | 0.36 |
| Extruded soybean | 10.00 | Lsy^2^ (%) | 1.40 |
| Fish meal | 1.00 | Met^2^ (%) | 0.40 |
| Whey powder | 2.00 | Thr^2^ (%) | 0.87 |
| Glucose | 2.80 |  |  |
| Soybean oil | 3.00 |  |  |
| Limestone | 1.15 |  |  |
| Dicalcium phosphate | 0.85 |  |  |
| Sodium chloride | 0.20 |  |  |
| Choline chloride | 0.08 |  |  |
| L-lysine HCl (98.5%) | 0.65 |  |  |
| DL-methionine | 0.11 |  |  |
| L-threonine | 0.20 |  |  |
| L-tryptophan | 0.03 |  |  |
| Premix^a^ | 1.50 |  |  |
| **Total** | **100.00** |  |  |

^a^: The premix provided the following per kilogram of complete diet: vitamin A 9,510 IU; vitamin D3 3,290 IU; vitamin E 50 IU; vitamin K3 4.5 mg; vitamin B1 3.5 mg; vitamin B2 6.7 mg; vitamin B6 3 mg; vitamin B12 0.03 mg; folic acid 1.2 mg; niacin 32.5 mg; pantothenic acid 28 mg; biotin 0.2 mg; iron 210 mg; copper 124 mg; zinc 1,600mg; manganese 58 mg; iodine 1.30 mg; and selenium 0.45 mg.^1^: Calculated values; ^2^: Analyzed values

The primary antibodies used for Western blot analysis

| **antibody** | **Catalog number, Dilution ratio, Company** |
| --- | --- |
| rabbit anti-β-actin | ab8227, 1:5000, Abcam plc, Cambridge, UK |
| mouse anti-TLR4 | 66350-1-Ig, 1:4000, Proteintech Group, Inc., Chicago, IL, USA |
| rabbit anti-IL-1β | 26028-1-AP, 1:3000, Proteintech Group, Inc., Chicago, IL, USA |
| mouse anti-JNK | 66210-1-Ig,1:10000, Proteintech Group, Inc., Chicago, IL, USA |
| rabbit anti-NF-κB | 80979-1-RR, 1:20000, Proteintech Group, Inc., Chicago, IL, USA |
| rabbit anti-p-JNK | 4668Sand, 1:10000, Proteintech Group, Inc., Chicago, IL, USA |
